# Arboreal mouse lemurs discovered sleeping in a burrow on the ground

**DOI:** 10.1002/ece3.9543

**Published:** 2022-12-04

**Authors:** Veronarindra Ramananjato, Finaritra Randimbiarison, Tanjoniaina Hery Nirina Patrick Rabarijaonina, Onja H. Razafindratsima

**Affiliations:** ^1^ Department of Integrative Biology University of California Berkeley Berkeley California USA; ^2^ Mention Zoologie et Biodiversité Animale University of Antananarivo, Faculty of Sciences Antananarivo Madagascar

**Keywords:** Cheirogaleidae, heterothermy, *Microcebus*, nesting, primates, tropical ecology

## Abstract

Finding sleeping sites is important for the fitness of many mammal species. Like most nonhuman primates, Madagascar's mouse lemurs (genus: *Microcebus*) are thought to exclusively use arboreal sleeping sites. The rufous mouse lemurs (*Microcebus rufus*) in Ranomafana National Park (southeastern Madagascar) have always been documented to sleep in either tree holes or leaf nests. However, in our recent field expedition, we observed, with the help of telemetry technologies, an unprecedented event of *M. rufus* sleeping in a burrow on the rainforest ground, curled up with a very slow heartbeat. Thus far, such behavior has not been observed in any other *Microcebus* species but is common in high‐altitude dwarf lemurs (genus: *Cheirogaleus*), a closely related genus to the mouse lemurs. We believe that this discovery could illustrate an ecophysiological response strategy to habitat changes, which warrants further investigation.

## Graphical abstract

We report here an unprecedented event of a mouse lemur sleeping in a burrow on the rainforest ground. We believe that this discovery could illustrate an ecophysiological response strategy to habitat changes.

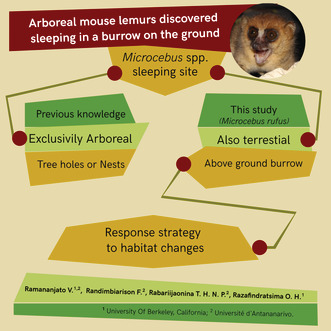

## Saro‐takarina

Zava‐dehibe ho an'ny biby mampinono ny toerana fatoriana izay miaro azy amin'ireo mpihaza azy sy miantoka ny fahaveloman'izy ireo izany, indrindra manoloana ny fiovaovan'ny toetrandro. Noeritreretina sy voalaza fa tsy mampiasa afa‐tsy fatoriana voajanahary anaty hazo ny Antsidy (taranaka: Microcebus; foko: Primates) eto Madagasikara. Eto kosa izahay dia mitatitra tranga sambany nisy nahita: Antsidy matory ao anaty lavaka tsy dia lalina amin'ny tany voasarona vatakazo. Tamin'ny alalan'ny onjam‐peo fanarahana azy ireo no nahitanay izany. Eritreretinay fa ity fampiasana lavaka amin'ny tany ity dia paikady entin'ny Antsidy hiarovan‐tena amin'ny hatsiaka, indrindra manoloana ny fiovana tampoka eo amin'ny firafitry ny toeram‐ponenany.

## 1 INTRODUCTION

Sleeping sites are crucial for mammal fitness, providing them significant support during their most vulnerable moments but can also be a limiting factor when habitats become degraded (Lutermann et al., 2010). Sleeping sites can also provide protection against predators. Because mammals spend a large portion of their lives resting and sleeping, they can be easily accessible to predators during such activities (Lima et al., 2005). Small mammals are often preferred as prey in tropical forests, making their vulnerability even increased when sleeping (Lutermann et al., 2010; Maher & Lott, 2000). Additionally, their small body size limits the quantity of fat they can support to maintain their body temperature constant (Lovegrove, 2005). The right sleeping site will thus provide them with insulation and reduce energy expenditure related to thermoregulation (Canale et al., 2012; Lovegrove, 2005). Therefore, documenting the type and quality of sleeping sites critical to determine the long‐term persistence of species in a changing world.

Many nonhuman arboreal primates have been reported to use arboreal sleeping sites, i.e., above the ground (Fruth et al., 2018). In Madagascar, this is even prominent because most lemurs are forest‐dwellers, thus they mainly use arboreal sleeping sites (Fruth et al., 2018; Mittermeier et al., 2010). Mouse lemurs (genus: *Microcebus*, family: Cheirogaleidae) are small‐bodied primates (body mass 24–90 g) and live in different forest ecosystems in Madagascar (Kappeler & Rasoloarison, 2003; Mittermeier et al., 2010). Eighty‐seven percent of mouse lemurs are currently threatened to extinction due to increasing habitat destruction and loss (Hending et al., 2022; Knoop et al., 2018). Mouse lemurs are considered solitary, but females change their grouping patterns around birth and nursing period (Blanco, 2008; Radespiel et al., 1998). Females live with their offspring in the same nest during the first months after birth, and a nest can shelter either one female and its offspring or multiple females and their respective offspring (Radespiel, 2006; Schülke & Ostner, 2005). They utilize the understory forest layer and are often observed to sleep/nest in tree holes or dense foliage (Karanewsky & Wright, 2015; Radespiel et al., 2003; Schmid, 1998). Such sleeping sites are critical for mouse lemurs because they are nocturnal. They become easy prey during the day when the predators are more active, thus they require protective sleeping sites (Fichtel, 2016). Additionally, their nocturnality implies a good thermoregulation strategy when facing the fluctuating temperatures of the day, which an insulated sleeping site can provide them (Karanewsky & Wright, 2015; Lutermann et al., 2010; Schmid, 1998). The choice of the adequate sleeping sites thus warrant their survival (Fichtel, 2016; Maher & Lott, 2000). To date, no studies have documented the possibility of mouse lemurs sleeping on the ground, similar to what was described as “unusual sleeping site” in larger‐bodied lemurs (e.g. Eppley et al., 2016). To our knowledge, no other mouse lemur species have used a burrow as a sleeping site; however, the dwarf lemurs (*Cheirogaleus* spp.), which are closely related to mouse lemurs, are known to hibernate underground (Blanco et al., 2013). Here, we report the first case of mouse lemurs sleeping in a burrow on the ground, thanks to radiotelemetry and active search.

## 2 MATERIALS AND METHODS

### 2.1 Study site and period

In June 2022, we conducted our fieldwork in Ranomafana National Park (RNP), southeastern Madagascar. RNP is an evergreen montane rainforest composed of native plant species under a tropical humid climate with an annual average temperature of 12–30°C and an annual average precipitation of 1500–2400 mm (Dunham et al., 2011). We sampled in Valohoaka (47°26′20.62″E, 21°17′48.52″S, 1182 m altitude), which is considered as a primary forest with relatively intact habitats (Ramananjato & Razafindratsima, 2021; Razafindratsima, 2017; Wright et al., 2012). During our expedition, the average temperature was 9–15°C with an average precipitation of 1200 mm (Centre Valbio, unpublished data).

### 2.2 Study species

**FIGURE 1:**
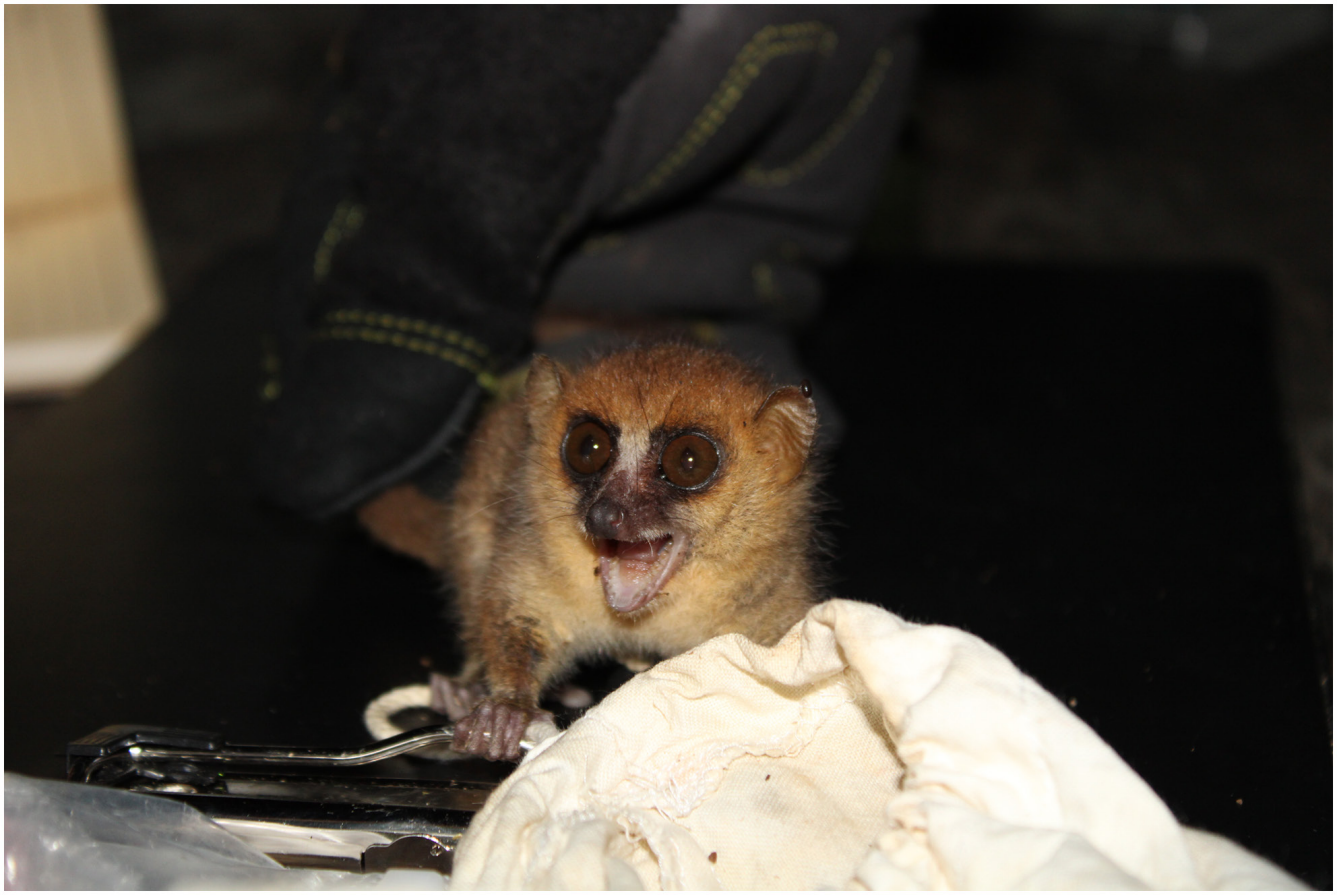
A grumpy female rufous mouse captured during the fieldwork expedition. This is one of the collared individuals of this study. Photo credit: Veronarindra Ramananjato

The only mouse lemur species in RNP is the rufous mouse lemur (*Microcebus rufus*; Figure 1) listed as Vulnerable on the IUCN Redlist of Threatened species (Louis et al., 2006; Wright et al., 2012, 2020). This species occurs at RNP with a density of 23 individuals/km^2^ (Wright et al., 2012, 2020). Individuals usually weigh 35–61 g and have an average body length of 12.5 cm (Mittermeier et al., 2010; V. Ramananjato, unpublished data). They use natural shelters such as tree holes and leaf nests at nearly 0.5–3 m above the ground as sleeping sites, in both intact and degraded forest habitats (Karanewsky & Wright, 2015; Knoop et al., 2018; Wright & Martin, 1995). Additionally, this species is known to enter into daily torpor during the coldest hours of the day, i.e., lethargic with very slow heartbeat (Atsalis, 1999, 2000; Blanco et al., 2018; Wright et al., 2012; Wright & Martin, 1995).

### 2.3 Radio‐tracking

We captured released mouse lemur individuals with passive live traps, following the protocol in Ramananjato et al. (2020). Over the study period, we equipped four adult individuals of *M. rufus* (two males and two females) with miniaturized radio‐transmitter collars (M1420, Advanced Telemetry Systems), following the protocol in Andriambeloson et al. (2021). Those individuals were torpid during capture release, so we did not use anesthesia to fit the collars around their neck. Collared individuals were monitored every hour at the campsite lab before their release at their capture location in the evening. We start the tracking 2 days after the release to give the individuals time to get used to the collars.

We tracked one collared individual per night from 6:00 to 10:00 p.m. for at least five nights per individual (nine tracking nights in total for the four individuals). We used the associated radio‐tracking device to locate the collared individuals. Once the device senses the transmitter, we proceed to an active search of the area, including all above‐ground natural shelters. We searched for the individual for about 2 h to make sure it is safe, and otherwise, retrieved the collar left in the sleeping site. Additionally, we restricted the frequency gain of the tracking device to a minimum and oriented the antenna to a different height to help us accurately locate the individual. Retrieved individuals are released the following dusk at their sleeping sites.

Ethical and legal approval was obtained prior to the start of the study. Animal handling and tracking were performed in accordance with the ethics of the permit from University of California Berkeley's Animal Care and Use Committee (AUP‐2021‐10‐14771) and the Ministère de l'Environnement et du Développement Durable of Madagascar (#428/21 and #137/22).

## 3 RESULTS

**FIGURE 2:**
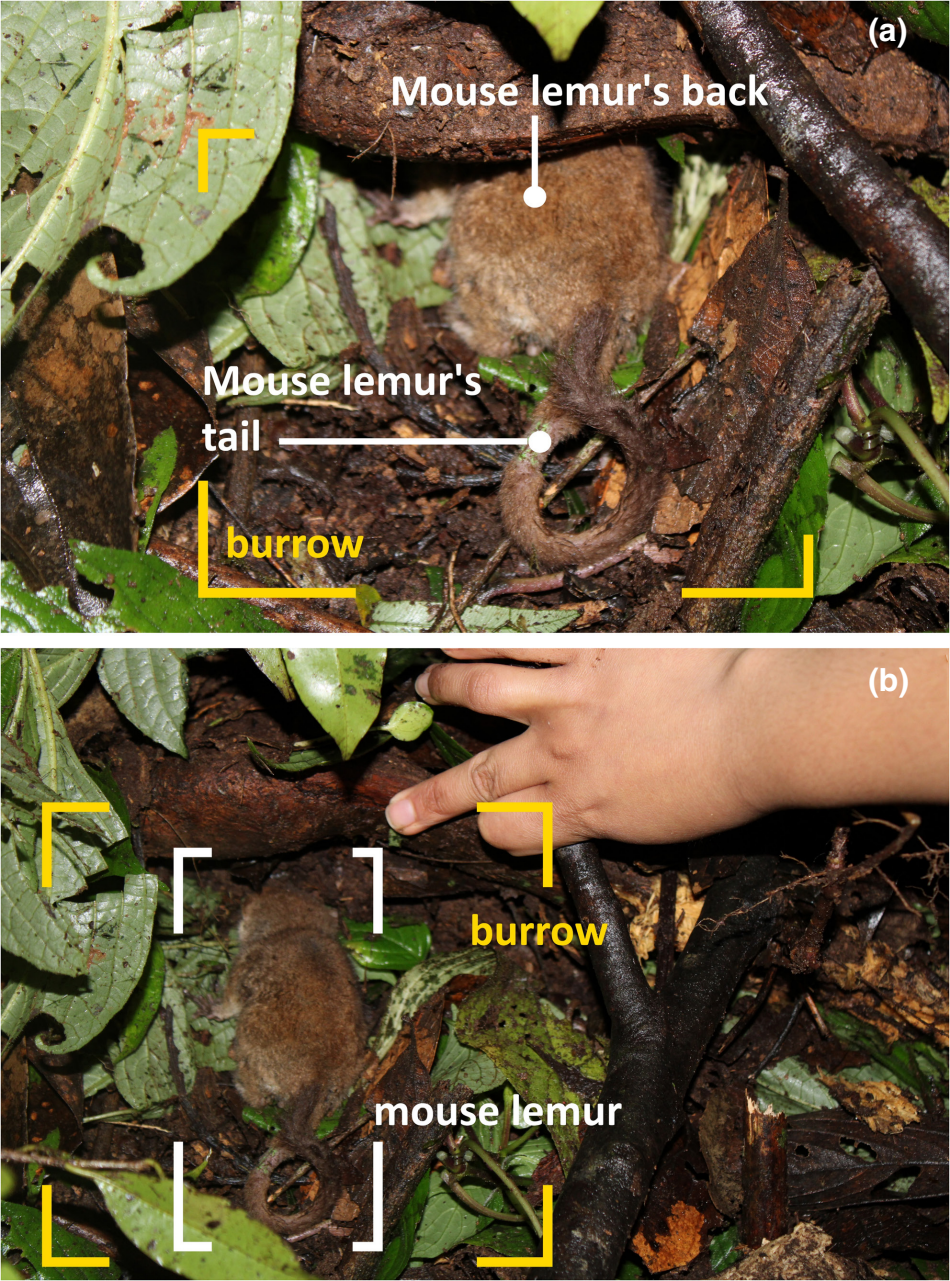
Torpid *Microcebus rufus* (a) found in a burrow on the ground and laying down on the leaf litter (b). Photo © Finaritra Randimbiarison

Over eight tracking nights, we located the sleeping sites of *M. rufus* on trees about 1.5–2 m high. The sleeping sites usually consisted of natural shelters such as tangled branches of understory trees, liana tangles, and dense foliage. For one tracking night, we located a torpid individual in a burrow on the ground (Figure 2).

The burrow consisted of one fallen log, under which the individual (body mass: 46 g, head‐body length = 88 mm, tail length = 108 mm, and tail circumference = 32 mm) was laying and resting on leaf litter (Figure 2). Growing herbaceous plants and leaf litter protected the burrow opening, through which we located the individual's back. The hole was 27.9 cm long and 8 cm deep (from the opening to the interior; Figure 2).

## 4 DISCUSSION

This study is the first report documenting *Microcebus rufus* using burrows on the ground as a sleeping site since they are known to interchangeably exploit tree holes and leaf nests (Karanewsky & Wright, 2015). To our knowledge, this is also the first report of such an event in *Microcebus* even despite the high variability in sleeping site selection in this genus (Karanewsky & Wright, 2015; Lutermann et al., 2010; Radespiel et al., 2003). Sheltering in non‐arboreal sleeping sites can be ecologically important for these small‐bodied animals that are often facing high predation pressure, fluctuating environmental conditions, and habitat disturbances.

Sleeping in a burrow on the ground can be an important strategy to avoid predators for an arboreal species. The natural predators of mouse lemurs (birds and snakes; Kappeler & Rasoloarison, 2003) might expect them to be in trees and thus may minimize foraging on the ground. However, by using burrows on the ground, mouse lemurs would be easily accessible to terrestrial predators such as Carnivora, which are known to forage primarily on the ground (Goodman, 2011) and directly in lemurs' nest (Deppe et al., 2008). Another explanation for such a choice of sleeping site is that the risk of predation from arboreal and terrestrial predators may be low or relaxed, similar to what Eppley et al. (2016) have suggested about the bamboo lemurs in Mandena, southeastern Madagascar. Sleeping on the ground can also be a good hibernaculum, i.e., a shelter used to hibernate during winter, which was the season of our study period. In general, *Microcebus* are known to use daily torpor (a shorter version of hibernation that usually lasts less than 24 h) during the dry season in response to low food availability and/or low environmental temperatures (Blanco et al., 2018; Canale et al., 2012). *Microcebus* species usually stay in tree holes or leaf nests, which buffer the fluctuating environmental temperatures and reduce the costs of thermoregulation (Schmid, 1998). Here, the dead log and the thick leaf litter on the ground could provide such insulating properties, limiting air exchanges between the individual and the environment (Eppley et al., 2016) and preventing rainwater to filter in. Finally, sleeping sites on the ground can be an acute response of the mouse lemurs to severe habitat disturbance. Between January and March 2022, three cyclones hit Madagascar and severely damaged the southeastern parts, including RNP (WFP, 2022; https://www.meteomadagascar.mg/). Many large trees have fallen (pers. obs.), knocking down smaller trees that mouse lemurs often prefer (Ramananjato & Razafindratsima, 2021), and may have reduced the number of tree holes available for mouse lemurs. The dead log on the burrow entrance may have come from such fallen trees and temporarily provide shelter for opportunistic animals such as the mouse lemurs, which are known to use degraded habitats as long as habitat structure and food are maintained (Knoop et al., 2018).

In summary, sleeping sites on the ground in mouse lemurs could indicate an ecophysiological strategy, a rapid adaptation to habitat disturbance, or both. Either way, this is an unprecedented finding that accentuates the various unknown aspects of the ecology of mouse lemurs, especially its ability to thrive in a changing world. Given the limitation of our observations, we were unable to test any hypothesis that could explain the use of terrestrial sleeping sites by *Microcebus*. Future research should, therefore, address such a topic, especially looking at the ecophysiological response strategies of *Microcebus* spp. and their close relatives. Understanding these strategies and their habitat use could provide critical information useful for drafting solutions for their conservation in the face of increasing natural and anthropogenic disturbances. Our observation may also open avenues for future research exploring the evolution of Malagasy primates.

## AUTHOR CONTRIBUTIONS

**Veronarindra Ramananjato:** Conceptualization (lead); data curation (lead); funding acquisition (lead); investigation (lead); methodology (lead); project administration (lead); validation (equal); writing – original draft (lead); writing – review and editing (lead). **Finaritra Randimbiarison:** Conceptualization (equal); data curation (equal); investigation (equal); methodology (equal); validation (equal); writing – original draft (equal); writing – review and editing (equal). **Tanjoniaina Hery Nirina Patrick Rabarijaonina:** Data curation (equal); investigation (equal); project administration (equal); validation (equal); writing – original draft (equal); writing – review and editing (equal). **Onja Razafindratsima:** Conceptualization (equal); funding acquisition (equal); methodology (equal); supervision (lead); validation (equal); writing – original draft (equal); writing – review and editing (equal).

## FUNDING INFORMATION

This article was funded by a Rufford Small Grant (#33285‐2), an African Graduate Research Fellowship from the American Society of Mammalogists, and World Wildlife Fund's Russell E. Train Faculty Fellowship (EF11883) to VR.

## CONFLICT OF INTEREST

The authors do not have competing or conflict of interests.

## Data Availability

Data sharing is not applicable to this article as no data sets were generated or analyzed during the current study.

## References

[ece39543-bib-0001] Andriambeloson, J. B. , Blanco, M. B. , Andriantsalohimisantatra, A. , Rivoharison, T. V. , Walker, N. , Birkinshaw, C. R. , & Yoder, A. D. (2021). Living in tiny fragments: A glimpse at the ecology of Goodman's mouse lemurs (*Microcebus lehilahytsara*) in the relic forest of Ankafobe, Central Highlands, Madagascar. Primates, 62, 887–896. 10.1007/s10329-021-00947-1 34541622

[ece39543-bib-0002] Atsalis, S. (1999). Diet of the brown mouse lemur *Microcebus rufus* in Ranomafana National Park, Madagascar. International Journal of Primatology, 20(2), 193–199. 10.1002/(SICI)1098-2345(200005)51:1<61::AID-AJP5>3.0.CO;2-2 10811440

[ece39543-bib-0003] Atsalis, S. (2000). Spatial distribution and population composition of the brown mouse lemur (*Microcebus rufus*) in Ranomafana National Park, Madagascar, and its implications for social organization. International Journal of Primatology, 51, 61–78. 10.1002/(SICI)1098-2345(200005)51:1<61::AID-AJP5>3.0.CO;2-2 10811440

[ece39543-bib-0004] Blanco, M. B. (2008). Reproductive schedule of female *Microcebus rufus* at Ranomafana National Park, Madagascar. International Journal of Primatology, 29, 323–338.

[ece39543-bib-0005] Blanco, M. B. , Dausmann, K. H. , Faherty, S. L. , & Yoder, A. D. (2018). Tropical heterothermy is cool: The expression of daily torpor and hibernation in primates. Evolutionary Anthropology, 27(4), 147–161. 30015414 10.1002/evan.21588

[ece39543-bib-0006] Blanco, M. B. , Daussmann, K. H. , Ranaivoarisoa, J. F. , & Yoder, A. D. (2013). Underground hibernation in a primate. Scientific Reports, 3, 1768. 10.1038/srep01768 23636180 PMC3641607

[ece39543-bib-0007] Canale, C. I. , Levesque, D. L. , & Lovegrove, B. G. (2012). Tropical heterothermy: Does the exception prove the rule or force a re‐definition? In Living a seasonal world—Thermoregulatory and metabolic adaptations (pp. 29–40). Springer‐Verlag.

[ece39543-bib-0008] Deppe, A. , Randriamiarisoa, M. , Kasprak, A. H. , & Wright, P. C. (2008). Predation on the brown mouse lemurs (*Microcebus rufus*) by a diurnal carnivore, the ring‐tailed mongoose (*Galidia elegans*). Lemur News, 13, 17–18.

[ece39543-bib-0009] Dunham, A. E. , Erhart, E. M. , & Wright, P. C. (2011). Global climate cycles and cyclones: Consequences for rainfall patterns and lemur reproduction in southeastern Madagascar. Global Change Biology, 17, 219–227.

[ece39543-bib-0010] Eppley, T. M. , Donati, G. , & Ganzhorn, J. U. (2016). Unusual sleeping site selection by southern bamboo lemurs. Primates, 57, 167–173. 10.1007/s10329-016-0516-4 26860934

[ece39543-bib-0011] Fichtel, C. (2016). Predation in the dark: Antipredator strategies of Cheirogaleidae and other nocturnal primates. In The dwarf and mouse lemurs of Madagascar (pp. 366–380). Cambridge University Press.

[ece39543-bib-0012] Fruth, B. , Tagg, N. , & Stewart, F. (2018). Sleep and nesting behavior in primates: A review. American Journal of Physical Anthropology, 166, 499–509. 10.1002/ajpa.23373 29989164

[ece39543-bib-0013] Goodman, S. M. (2011). Les Carnivora de Madagascar, Guide sur la diversité biologique de Madagascar. Association Vahatra.

[ece39543-bib-0014] Hending, D. , Holderied, M. , McCabe, G. , & Cotton, S. (2022). Effects of future climate change on the forests of Madagascar. Ecosphere, 13(4), e4017.

[ece39543-bib-0015] Kappeler, P. M. , & Rasoloarison, R. M. (2003). *Microcebus*, Mouse lemur, Tsidy. In The natural history of Madagascar (pp. 1310–1315). University of Chicago Press.

[ece39543-bib-0016] Karanewsky, C. J. , & Wright, P. C. (2015). A preliminary investigation of sleeping site selection and sharing by the brown mouse lemur *Microcebus rufus* during the dry season. Journal of Mammalogy, 96, 1344–1351. 10.1093/jmammal/gyv143

[ece39543-bib-0017] Knoop, S. , Chikhi, L. , & Salmona, J. (2018). Mouse lemurs' use of degraded habitat: A review of the literature. Lemur News, 21, 20–31.

[ece39543-bib-0018] Lima, S. L. , Rattenborg, N. C. , Lesku, J. A. , & Amlaner, C. J. (2005). Sleeping under the risk of predation. Animal Behaviour, 70, 723–736. 10.1016/j.anbehav.2005.01.008

[ece39543-bib-0019] Louis, E. E. , Coles, M. S. , Andriantompohavana, R. , Sommer, J. A. , Engberg, S. E. , Zaonarivelo, J. R. , Mayor, M. I. , & Brenneman, R. A. (2006). Revision of the mouse lemurs (*Microcebus*) of eastern Madagascar. International Journal of Primatology, 27(2), 347–389.

[ece39543-bib-0020] Lovegrove, B. G. (2005). Seasonal thermoregulatory responses in mammals. Journal of Comparative Physiology B, 175, 231–247. 10.1007/s00360-005-0477-1 15900504

[ece39543-bib-0021] Lutermann, H. , Verburgt, L. , & Rendigs, A. (2010). Resting and nesting in small mammal: Sleeping sites as a limiting resource for female grey mouse lemurs. Animal Behavior, 79(6), 1211–1219.

[ece39543-bib-0022] Maher, C. R. , & Lott, D. F. (2000). A review of ecological determinants of territeriality within vertebrate species. The American Midland Naturalist, 143, 1–29.

[ece39543-bib-0023] Mittermeier, R. A. , Louis, E. E. , Richardson, M. , Schwitzer, C. , Langrand, O. , & Rylands, A. (2010). Lemurs of Madagascar (3rd ed.). Conservation International‐Tropical Field Guide Series.

[ece39543-bib-0024] Radespiel, U. (2006). Ecological diversity and seasonal adaptations of mouse lemurs (Microcebus spp.). In L. Gould & M. L. Sauther (Eds.), Lemurs, developments in primatology: Progress and Prospect (pp. 211–234). Springer US. 10.1007/978-0-387-34586-4_10

[ece39543-bib-0025] Radespiel, U. , Cepok, S. , Zietemann, V. , & Zimmerman, E. (1998). Sex‐specific usage patterns of sleeping sites in grey mouse lemurs *Microcebus murinus* . American Journal of Primatology, 46, 77–84. 9730214 10.1002/(SICI)1098-2345(1998)46:1<77::AID-AJP6>3.0.CO;2-S

[ece39543-bib-0026] Radespiel, U. , Ehresmann, P. , & Zimmermann, E. (2003). Species‐specific usage of sleeping sites in two sympatric mouse lemur species (*Microcebus murinus* and *M. ravelobensis*) in northwestern Madagascar. American Journal of Primatology, 59, 139–151. 10.1002/ajp.10071 12682922

[ece39543-bib-0027] Ramananjato, V. , Rakotomalala, Z. , Park, D. S. , DeSisto, C. M. M. , Raoelinjanakolona, N. N. , Guthrie, N. K. , Fenosoa, Z. E. S. , Johnson, S. E. , & Razafindratsima, O. H. (2020). The role of nocturnal omnivorous lemurs as seed dispersers in Malagasy rain forests. Biotropica, 52(4), 758–765. 10.1111/btp.12789

[ece39543-bib-0028] Ramananjato, V. , & Razafindratsima, O. H. (2021). Structure of microhabitats used by *Microcebus rufus* across a heterogeneous landscape. International Journal of Primatology, 42, 682–700. 10.1007/s10764-021-00224-4

[ece39543-bib-0029] Razafindratsima, O. H. (2017). Post‐dispersal seed removal by rodents in Ranomafana rain forest, Madagascar. Journal of Tropical Ecology, 33, 232–236.

[ece39543-bib-0030] Schmid, J. (1998). Tree holes used for resting by gray mouse lemurs (*Microcebus murinus*) in Madagascar: Insulation capacities and energetic consequences. International Journal of Primatology, 19(5), 797–809.

[ece39543-bib-0031] Schülke, O. , & Ostner, J. (2005). Big times for dwarfs: Social organization, sexual selection, and cooperation in the Cheirogaleidae. Evolutionary Anthropology, 14, 170–185.

[ece39543-bib-0032] WFP . (2022). WFP Madagascar: Cyclone response update, WFP Madagascar. ed. World Food Program.

[ece39543-bib-0033] Wright, P. C. , Erhart, E. M. , Tecot, S. , Baden, A. L. , Arrigo‐Nelson, S. J. , Herrera, J. , Morelli, T. L. , Blanco, M. B. , Deppe, A. , Atsalis, S. , Johnson, S. , Ratelolahy, F. , Tan, C. , & Zohdy, S. (2012). Long‐term lemur research at Centre Valbio, Ranomafana National Park. In Long‐term field studies of primates (pp. 67–100). Springer‐Verlag.

[ece39543-bib-0034] Wright, P. C. , Hearthstone, E. , Zakamanana, F. , Andrianoely, D. , & Donohue, M. E. (2020). Microcebus rufus.

[ece39543-bib-0035] Wright, P. C. , & Martin, L. B. (1995). Predation, pollination and torpor in two nocturnal prosimians: *Cheirogaleus major* and *Microcebus rufus* in the rain forest of Madagascar. In Creatures of the dark: The nocturnal prosimians (pp. 45–60). Plenum Press.

